# Early Positive Approaches to Support (E-PAtS) for Families of Young Children With Intellectual Disability: A Feasibility Randomised Controlled Trial

**DOI:** 10.3389/fpsyt.2021.729129

**Published:** 2021-12-21

**Authors:** Elinor Coulman, Nick Gore, Gwenllian Moody, Melissa Wright, Jeremy Segrott, David Gillespie, Stavros Petrou, Fiona Lugg-Widger, Sungwook Kim, Jill Bradshaw, Rachel McNamara, Andrew Jahoda, Geoff Lindsay, Jacqui Shurlock, Vaso Totsika, Catherine Stanford, Samantha Flynn, Annabel Carter, Christian Barlow, Richard P. Hastings

**Affiliations:** ^1^Centre for Trials Research, Cardiff University, Cardiff, United Kingdom; ^2^Tizard Centre, University of Kent, Cornwallis North East, Canterbury, United Kingdom; ^3^Nuffield Department of Primary Care Health Sciences, University of Oxford, Oxford, United Kingdom; ^4^Institute of Health and Wellbeing, University of Glasgow, Glasgow, United Kingdom; ^5^Centre for Educational Development, Appraisal and Research, University of Warwick, Coventry, United Kingdom; ^6^The Challenging Behaviour Foundation, Kent, United Kingdom; ^7^Division of Psychiatry, Faculty of Brain Sciences, University College London, London, United Kingdom; ^8^Centre for Developmental Psychiatry and Psychology, Monash University, Melbourne, VIC, Australia

**Keywords:** intellectual disability, developmental disability, developmental delay, randomised controlled trial, parenting, Early Positive Approaches to Support (E-PAtS), mental well-being, support

## Abstract

**Background:** Parents of children with intellectual disabilities are likely to experience poorer mental well-being and face challenges accessing support. Early Positive Approaches to Support (E-PAtS) is a group-based programme, co-produced with parents and professionals, based on existing research evidence and a developmental systems approach to support parental mental well-being. The aim of this study was to assess the feasibility of community service provider organisations delivering E-PAtS to parents/family caregivers of young children with intellectual disability, to inform a potential definitive randomised controlled trial of the effectiveness and cost-effectiveness of E-PAtS.

**Methods:** This study was a feasibility cluster randomised controlled trial, with embedded process evaluation. Up to two parents/family caregivers of a child (18 months to <6 years old) with intellectual disability were recruited at research sites and allocated to intervention (E-PAtS and usual practise) or control (usual practise) on a 1:1 basis at cluster (family) level. Data were collected at baseline and 3 and 12 months' post-randomisation. The following feasibility outcomes were assessed: participant recruitment rates and effectiveness of recruitment pathways; retention rates; intervention adherence and fidelity; service provider recruitment rates and willingness to participate in a future trial; barriers and facilitating factors for recruitment, engagement, and intervention delivery; and feasibility of collecting outcome measures.

**Results:** Seventy-four families were randomised to intervention or control (*n* = 37). Retention rates were 72% at 12 months post-randomisation, and completion of the proposed primary outcome measure (WEMWBS) was 51%. Recruitment of service provider organisations and facilitators was feasible and intervention implementation acceptable. Adherence to the intervention was 76% and the intervention was well-received by participants; exploratory analyses suggest that adherence and attendance may be associated with improved well-being. Health economic outcome measures were collected successfully and evidence indicates that linkage with routine data would be feasible in a future trial.

**Conclusions:** The E-PAtS Feasibility RCT has demonstrated that the research design and methods of intervention implementation are generally feasible. Consideration of the limitations of this feasibility trial and any barriers to conducting a future definitive trial, do however, need to be considered by researchers.

**Clinical Trial Registration:** https://www.isrctn.com, identifier: ISRCTN70419473.

## Background

Parents of children with intellectual disabilities (ID) experience elevated levels of stress and mental health problems. Mothers of children with ID are more likely than other mothers to experience depression (1) and psychological distress, as measured on a mental health screening questionnaire (2). Fathers of children with ID are twice as likely as other fathers to score above the cut-off on a psychiatric disorder screen in a representative population-based sample (3). Furthermore, parents of children with ID report a higher care burden load and greater levels of financial difficulties (4). Well-being of parents and other family members, has been shown to be inversely related to behavioural and emotional problems in children with ID, and vice versa (5). There are also wider implications for other family members and family functioning as a whole, with parental, parent-child, and sibling relationships potentially being adversely affected (5). Despite the evident need, parents of young children with ID report that access to appropriate support is limited (6).

A number of parenting programmes have been shown to be potentially effective at improving well-being of parents whose children have ID. However, these have typically been adapted from existing parenting programmes that have a main focus on improving children's behaviour problems (7, 8) rather than developed specifically for parents of children with ID and with broader developmental outcomes in mind. Of 15 studies concerning (parenting) programmes reviewed by the National Institute of Clinical Excellence (9) only one (10) had been developed specifically for families of children with ID (rather than through adaptation from a mainstream programme). Furthermore, none of these programmes had the primary aim of improving parent psychosocial well-being. A further limitation of existing parenting programmes is that few have utilised co-production methods that involve family members in intervention design or development. Finally, most existing programmes were not developed specifically in the context of the early years of development (i.e., under 5 years of age) for early intervention with families of children with intellectual disability.

In response to the identified gaps in the evidence base, the Early Positive Approaches to Support (E-PAtS) programme was developed. E-PAtS was designed specifically as a group programme for families of young children with ID (i.e., as an early intervention) with a primary focus on enhancing parental psychosocial well-being in the context of caregiving. It is informed by ID research evidence and co-produced with family carers and professionals.

The aim of the feasibility randomised controlled trial (RCT) was to assess the feasibility of delivering E-PAtS to parents/caregivers of children with ID by community parenting support service provider organisations. The study will inform a potential, definitive RCT of the effectiveness and cost-effectiveness of E-PAtS. The main objectives of the feasibility (RCT) were to assess: (1) the feasibility of recruiting eligible participants, and identifying the most effective recruitment pathways to identify families of young children with ID; (2) feasibility of recruiting suitable service provider organisations and facilitators to deliver the E-PAtS intervention; (3) recruitment rates and retention through 3 and 12 months' post-randomisation follow-up data collection; (4) the acceptability of study processes, including randomisation, to service provider organisations, facilitators and family caregivers, assessed through qualitative interviews; (5) acceptability of intervention delivery to service provider organisations, facilitators and family caregivers; (6) adherence to the intervention, and reach and fidelity of implementation of the E-PAtS intervention; (7) usual practise in this setting and use of services/support by intervention and control participants; (8) the feasibility and acceptability of proposed outcome measures for a definitive trial, including resource use and health-related quality of life data, as methods for measuring the effectiveness and cost-effectiveness of the intervention and for conducting an embedded health economic evaluation within a definitive randomised controlled trial (RCT); (9) the acceptability of collecting and analysing routine data within a definitive RCT; and (10) service provider organisation willingness to participate in a definitive trial.

## Methods

Full methods are detailed by Coulman et al in the peer-reviewed protocol article (11). A brief description follows.

### Participant Selection and Randomisation

Service provider organisations (organisations who offer support services to parents of children with ID) were selected as sites if they: (1) were prepared to refer a sufficient number of potential families to the trial team and (2) were prepared to deliver E-PAtS immediately and following 12 months post-randomisation. Families were referred to the trial team from three sites (ID charity Mencap) in England and Northern Ireland via a multi-point recruitment method, consisting of established referral routes; local and national charitable support organisations; local authority services; special schools and nurseries; after school/weekend services for children with special educational needs and disabilities; parent/family support groups; social media; advertising in the media in local areas; and self-referral. Up to two members (main family carer and second family carer) from each family could participate. Eligibility was established during a telephone or face-to-face meeting with a researcher; screening criteria items and screening measures, including the Vineland Adaptive Behaviour Scales (Third Edition) (Vineland-3) (12) and the Brief Family Distress Scale (13), were completed.

Participants were eligible for the trial if they were ≥18 years old, a biological, step, adoptive, foster (if placement was planned to last until 12 months' post-randomisation) or other family caregiver of a child (aged 18 months to 5 years-up to the day before the child's 6th birthday), who had an administrative label of any severity of ID and had an Adaptive Behaviour Composite (ABC) score on the Vineland-3 of <80. The main family carer had to be available to attend the E-PAtS group sessions and have a sufficient level of spoken English to complete the outcome measures. Participants were excluded if there were any child protection concerns identified, if the main family carer was enrolled in a group or individually delivered parenting programme or programme of personal therapeutic support, or if the family was in a state of current crisis (measured as a score of 9 or 10 on the Brief Family Distress Scale) (13).

Participants were informed about the trial and provided consent to participate. To assess whether a waitlist intervention would be desirable for control participants in a definitive trial, participants were asked to select their choice of either trial pathway A (control participants offered E-PAtS on a waitlist) or pathway B (control participants not offered E-PAtS as part of the trial). Participants selected their preference for method of follow-up data collection (telephone, face-to-face, or postal) and completed baseline outcome measures. Families were randomised in a 1:1 ratio to intervention plus usual practise (UP) or to UP alone, using randomly permuted blocks (block size 4), stratified by trial site and choice of trial pathway (A or B). Researchers remained blind to allocation, with any incidents of unblinding being recorded. Participants were followed up at 3 and 12 months' post-randomisation, and outcome measure data were collected via the participants' preferred data collection method.

### Early Positive Approaches to Support (E-PAtS) Intervention

E-PAtS is a group support programme for parents and family carers of young children (5 years of age and under) with ID; and so has been designed explicitly as an early intervention. It aims to improve outcomes for both parents/family carers and their children with ID, as well as other family members. E-PAtS was co-produced by family carers and professionals and informed by relevant research evidence and by early intervention theory such as the Developmental Systems Model (14).

E-PAtS is a fully manualised programme, co-delivered by a family carer facilitator and professional facilitator (either health or social care) dyad. Family career and professional facilitators received training together in E-PAtS from the programme developers in small groups following a 5 day course including theoretical and evidence background, intervention content, and the opportunity to practise sections of the training with peers and to receive feedback.

E-PAtS comprises: (1) an individual, supportive preparatory interview with the co-facilitator or a representative from their organisation, with the aim of supporting attendance and engagement; (2) eight (typically weekly) 2.5 hourly group sessions, delivered in community settings, and (3) a personalised accompanying workbook and associated resources that can be completed throughout the programme, and that also facilitate dissemination of information to other family members and supporters. Each group session focuses on supporting parents'/family carers' well-being and behaviour in the context of parenting a child with ID and provides evidence-based content in the form of oral and video presentations, structured exercises, and group discussion.

Adherence to the E-PAtS intervention was defined as at least one family carer from a family attending five out of eight intervention sessions. An overview of the E-PAtS group session content is presented in Table 1. All sessions were designed to have a positive focus (hence “positive support”) about the strengths and experiences of families that can be useful for other families, and also having an optimistic orientation. Families face challenges when they have a young child with ID, but positive solutions are available to help build the child's skills and improve quality of life for families. Overall, each session includes some psycho-educational elements (e.g., explaining sleep cycles) and some practical strategies that can be used with young children with ID–informed by the existing evidence base and also families' successful strategies (i.e., as a part of the original co-production process).

**Table 1:** Content of the Early Positive Approaches to Support (E-PAtS) Programme sessions.

**Session 1**
Working together	• Establishing a socially and emotionally supportive group • Orientation and key messages about the Programme • Information and strategies to support access to support services
**Session 2**
Looking after you and your family	•Key information about well-being • Maximising social and emotional therapeutic group processes • Developing proactive well-being strategies • Developing emotional coping strategies • Information and signposting to well-being supports/services
**Session 3**
Supporting sleep	•Key information about sleep and sleep difficulties • Development of bespoke sleep strategies for children of group members • Family carers well-being in the context of supporting a child's sleep • Information and signposting to sleep supports/services
**Session 4**
Interaction and communication	•Key information about communication development and communication difficulties • Development of bespoke strategies to support receptive and expressive communication partnerships • Family carers well-being in the context of supporting a communication for a child • Information and signposting to communication supports/services
**Session 5**
Supporting active development	•Key information about engagement in activity and adaptive skill development • Establishing core strategies to support activity engagement and skill development for individual children • Family carers well-being in the context of supporting an engagement and skill development for a child • Information and signposting to relevant supports/services
**Session 6**
Supporting challenges 1	•Key information about development and maintenance of behaviours that challenge • Identification of core proactive strategies that can support life quality and reduce risk of behaviours that challenge for group member's children • Family carers well-being in the context of supporting a behaviour that challenges for a child • Information and signposting to relevant supports/services
**Session 7**
Supporting challenges 2	•Key information about episodes of behaviours that challenge and corresponding support needs of children • Strategies to support understanding of a behaviour that challenges for an individual child and establishment of bespoke reactive and proactive behavioural supports • Family carers well-being in the context of supporting behaviour that challenges for a child • Information and signposting to relevant supports/services
**Session 8**
Bringing it all together	•Integration of all concepts, strategies and discussions • Development of future plans for individual group members to support themselves and their family • Opportunities to provide feedback and contribute to the co-production of future programme delivery • Socially and emotionally supportive group processes to support end of programme • Information and signposting to relevant supports/services

### Measures

#### Trial Feasibility Outcomes

Feasibility was the primary outcome in this trial. The following feasibility outcomes, measured using a combination of descriptive, quantitative, and qualitative data, informed the decision to progress to a later definitive trial: (1) recruitment rates and effectiveness of recruitment pathways; (2) retention rates; (3) Adherence to the E-PAtS programme; (4) Fidelity of the delivery of the E-PAtS programme; (5) Service provider organisation recruitment rates and willingness to participate in feasibility and definitive trial; (6) Assessment of the barriers and facilitating factors for recruitment; engagement, and intervention delivery from the perspective of all stakeholders; (8) usual practise in this setting and use of services/support by intervention and control participants; (9) Feasibility of collecting and analysing routine collected data within a definitive trial; and (10) feasibility and acceptability of collecting and analysing proposed outcome measures including those required to conduct a health economic evaluation within a future definitive trial.

#### Participant-Reported Outcome Measures

At baseline, participants provided demographic data for the child (gender, date of birth, ID and any other related diagnoses, education setting, and living arrangements) and carer (ethnicity, qualifications, employment status, financial resource questions, and relationship to child). The feasibility of using a range of outcome measures, proposed to assess the effectiveness of the E-PAtS programme in a later definitive RCT, was assessed. Table 2 details all outcome measures that were collected at baseline and follow-ups. The measures were chosen because of their match with the underpinning Logic Model for E-PAtS in terms of change processes and potential short, medium, and longer term outcomes. The questionnaire package took up to an hour to complete at each data collection point.

**Table 2:** Timings of outcome measures.

	**Baseline**		
**Timepoint**	**Up to 8 weeks prior to randomisation**	**3 months post-randomisation**	**12 months post-randomisation**
Vineland adaptive behaviour scales (VABS) (3rd) FULL (12).	X		
Brief family distress scale (13)	X		
Demographic data	X		
Warwick-Edinburgh mental well-being scale (15)	X	X	X
Hospital anxiety and depression scale (16)	X	X	X
EQ-5D-5L (17)	X	X	X
Brief COPE (18)	X	X	X
Child behaviour checklist (CBCL) (19)	X	X	X
Paediatric quality of life inventoryTM version 4.0 generic core scales (20)	X	X	X
Happiness of relationship scale (21)	X	X	X
Family APGAR scale (22)	X	X	X
Strengths and difficulties questionnaire (23)	X	X	X
Sibling relationship questionnaire (revised) (where relevant) (24)	X	X	X
Family support scale (25)	X	X	X
5 min speech sample (26)	X	X	X
Parenting sense of competence scale (7 items) (27)	X	X	X
Positive gains scale (28, 29)	X	X	X
Disagreement over issues related to child (21), co-parenting (30)	X	X	X
Child-parent relationship scale (31)	X	X	X
Parent activities/involvement index	X	X	X
Group cohesion scale (8 items) (32)		X	
Client service receipt inventory (33)	X	X	X
Vineland adaptive behaviour scales (VABS) (3rd) brief (12)			X
Participant views on use of routine collected data in future trial			X

### Process Evaluation

A process evaluation, informed by MRC guidance (34), addressed key trial objectives using a mixed methods approach. Qualitative interviews with facilitators (who delivered the intervention), service provider organisations, and parents/family carers explored the feasibility of the research design and implementation in the context of implementing E-PAtS within a definitive trial. All facilitators and service providers were invited to an interview and parents/family carers were purposively sampled to ensure a representative spread across sites, randomisation allocation, family carer status (main family carer vs. second family carer) and attendance levels at the group sessions. Quantitative methods included the assessment of recruitment rates/patterns, attendance and intervention fidelity, reach, and adherence. Fidelity of intervention delivery was assessed by completion of two checklists, consisting of items that correspond to whether key activities and discussions were completed in a given group session: (1) completion of E-PAtS Observation Checklist by a trained observer based on video recorded or audio recorded sessions; and (2) self-completion of facilitator implementation checklists following each session. For both checklists, items were scored as present (1) or missing (0).

### Data Analyses

#### Statistical Analysis

As this was a feasibility trial, no hypothesis testing was performed (34). Descriptive statistics were reported as means and standard deviations or medians and interquartile ranges, as appropriate, and categorical data reported as frequencies and proportions. All statistical analysis was carried out using Stata version 16.1.

To estimate the mean difference between intervention and control groups for the proposed primary outcome measure for a definitive trial [the Warwick Edinburgh Mental Well-Being Scales (WEMWBS) at 12-months post-randomisation], two-level linear regression models (accounting for clustering of family carers within families) were fitted, adjusting for baseline WEMWBS score, trial site, and choice of pathway. All families in the groups to which they were randomised were analysed, regardless of intervention receipt. Mean differences were reported alongside 95% confidence intervals. Remaining potential outcome measures for a definitive trial were analysed with appropriate multilevel regression models and reported using point estimates and 95% confidence intervals.

To explore differences between arms after accounting for intervention receipt, complier average causal effect (CACE) analyses were conducted for the adherence definition and two measures of session attendance [(1) actual number of sessions attended by a family unit (i.e. at least one family member) and (2) actual number of sessions attended by the main family carer], by fitting two-stage least squares instrumental variables regression models. Models included baseline WEMWBS scores and site as covariates and accounted for the correlated nature of participants within families by including cluster robust standard errors. Estimates are reported as adjusted mean differences and associated 95% confidence intervals. For session attendance, model coefficients were multiplied by eight (i.e., the maximum number of sessions which could have been received) to estimate the maximum efficacy.

#### Economic Analysis

The aim of the economic analysis was to assess the most appropriate ways to measure and express the cost-effectiveness of the programme within a later definitive trial. As such, the following were completed: (1) All associated costs to deliver each E-PAtS group session were reported by facilitators in weekly logs, and facilitator employer costs, including salaries, employer on costs and revenue, and capital overheads, were collected to estimate the costs of delivering E-PAtS in community settings; (2) The feasibility of collecting the broader resource use and health-related quality outcomes associated with E-PAtS was assessed; (3) Appropriate sources of unit costs for potential resource consequences were identified; (4) Routine data sources were identified that would allow extraction of health and social care data to complement and validate self-reported resource use data; and (5) The most appropriate ways for expressing the cost-effectiveness of the E-PAtS programme was determined.

#### Routine Data Analysis

Whilst not collecting routine data specifically for this trial, due to ethical and cost implications, the trial aimed to identify routine data sources to be used for a later definitive trial and explore the feasibility and acceptability of collecting routine data in a definitive trial through participant-reported quantitative data and participant interviews.

#### Qualitative Analysis

Each set of interviews [participants (*n* = 30), facilitators (*n* = 8), service provider organisations (*n* = 2)] were analysed separately and independently using thematic analysis. A sample was reliability checked by an independent researcher. Qualitative synthesis all of themes across all subsets of interviews provided an over-arching synthesis of parent/family carers' views and experiences (35). Qualitative and quantitative data analysis results were then combined by triangulation.

### Public Involvement

Public involvement in this trial included: (1) Trial Steering Committee (TSC) contribution by a family carer; and (2) contribution, *via* face-to-face or virtual meetings and email contacts, by a Family Carer Advisory Group, consisting of nine parents/family carers, managed by the Challenging Behaviour Foundation (CBF). The remit of the Family Carer Advisory Group included advising the trial team on: facilitator role advertising, parent/family carer-facing documentation including recruitment materials and wording of questionnaires/outcome measures, qualitative interview topic guides, reports/written documentation, and dissemination of trial findings and future research priorities.

## Results

### Feasibility of Recruiting Eligible Participants, Most Effective Recruitment Pathways, and Recruitment Rates

Of 150 families who expressed an interest in participating, 88 were screened and 74 consented, randomised and completed baseline measures (95 participants), representing a 50% recruitment rate. Participants were recruited across three sites in two recruitment phases (26th March−18th May 2018 and 15th June−13th August 2018). An additional recruitment period was required to meet the target, due to differences in school term times in one of the sites recruited to take part in the feasibility RCT. Site differences in recruitment rates were observed; site 1, 2, and 3 recruited 38, 22, and 14 families, respectively. Participant/family referral directly *via* the service provider organisation (ID charity Mencap) was the most successful referral route with 92.7% (*n* = 139) of families referred in this way. Only 7.3% (*n* = 11) of families were referred indirectly *via* advertising or word of mouth. Interviewed facilitators (*n* = 8) suggested that the methods used to identify potentially eligible families were feasible to implement. Facilitators reported that randomisation (and therefore possible allocation to the control group) was seen as a barrier to participation when explaining the trial to family carers, but not to the extent that it appeared to undermine the feasibility or acceptability of a full randomised controlled trial.

Interviewed participants' (*n* = 30) main motivation to participate was to support their child with ID and to be able to deal more effectively with specific challenges. Other reasons included altruism, to both help future families and support research, due to the perceived lack of support currently available. The majority of main family carers were biological mothers (*n* = 65, 88%) with only four biological fathers (5%), whilst the majority of second family carers (*n* = 21) were biological fathers (*n* = 18, 86%). Participant characteristics, as well as the characteristics of their children with ID, were broadly balanced between trial arms. Separate parent/family carer and child demographic data for both arms of the trial are presented in Tables 3, 4.

**Table 3:** Participant demographics split by trial arm and family carer status.

	**Main family carer**		**Second family carer**	
	**Control (*n* = 37)**	**Intervention (*n* = 37)**	**Control (*n* = 10)**	**Intervention (*n* = 11)**
	***n* (%)**	***n* (%)**	***n* (%)**	***n* (%)**
**Relationship to child**				
Biological mother	30 (81)	35 (95)	0 (0)	0 (0)
Biological father	4 (11)	0 (0)	9 (90)	9 (82)
Adoptive mother	1 (3)	0 (0)	0 (0)	0 (0)
Adoptive father	0 (0)	0 (0)	1 (10)	0 (0)
Foster mother	0 (0)	2 (5)	0 (0)	0 (0)
Grandmother	0 (0)	0 (0)	0 (0)	2 (18)
Missing	2 (5)	0 (0)	0 (0)	0 (0)
**Living arrangements**				
Child lives with family full-time	35 (95)	34 (92)	10 (100)	7 (64)
Child lives with family part-time	(0)	1 (3)	0 (0)	3 (27)
Missing	2 (5)	2 (5)	0 (0)	1 (9)
**Ethnicity**				
Black/African/Black British: African/Caribbean/other	3 (8)	6 (16)	0 (0)	1 (9)
Mixed other	0 (0)	1 (3)	0 (0)	0 (0)
Ethnic other	1 (3)	0 (0)	0 (0)	0 (0)
White: English/Welsh/Scottish/Northern Irish/British/Irish/Other	30 (81)	26 (71)	10 (100)	10 (91)
Any other ethnic background	1 (3)	1 (3)	0 (0)	0 (0)
Prefer not to say	0 (0)	1 (3)	0 (0)	0 (0)
Missing	2 (5)	2 (5)	0 (0)	0 (0)
**Qualifications**				
No qualifications	2 (5)	0 (0)	0 (0)	1 (9)
Some GCSEs passes or equivalent	5 (14)	5 (14)	2 (20)	3 (27)
5 or more GCSEs at A–C or equivalent	3 (8)	4 (11)	1 (10)	1 (9)
5 A/AS Levels or equivalent	0 (0)	2 (5)	0 (0)	0 (0)
Higher Education but below degree level	10 (27)	7 (19)	2 (20)	2 (18)
Degree (e.g., BA, BSC, MA)	14 (38)	17 (46)	5 (50)	3 (27)
Don't know	1 (3)	0 (0)	0 (0)	0 (0)
Missing	2 (5)	2 (5)	0 (0)	1 (9)

**Table 4:** Baseline characteristics of child with ID.

	**Reported by main family carer**	
	**Control (*n* = 37)**	**Intervention (*n* = 37)**
	**Number (%)**	**Number (%)**
**Gender of child**		
Male	23 (62)	27 (73)
Female	12 (32)	10 (27)
Missing	2 (5)	0 (0)
**School/nursery attendance**		
Not in school/nursery	10 (27)	14 (38)
Mainstream preschool/nursery	9 (24)	5 (14)
SRB in mainstream preschool/nursery	4 (11)	3 (8)
Mainstream school	1 (3)	3 (8)
Special school	2 (5)	1 (3)
Special preschool/nursery	8 (22)	9 (24)
Missing	3 (8)	2 (5)
**Visual impairment**		
No	26 (70)	26 (70)
Yes	9 (24)	8 (22)
Missing	2 (5)	3 (8)
**Hearing impairment**		
No	29 (78)	30 (81)
Yes	6 (16)	4 (11)
Missing	2 (5)	3 (8)
**Physical health problems**		
No	19 (51)	20 (54)
Yes	16 (43)	13 (35)
Missing	2 (5)	4 (11)
**Sibling aged 4–16**		
No	10 (27)	14 (38)
Yes	25 (68)	22 (59)
Missing	2 (5)	1 (3)

### The Feasibility of Recruiting Suitable Service Provider Organisations and Facilitators to Deliver the E-PAtS Intervention

All recruited sites (*n* = 3) were third sector organisations supporting families of children with ID, two of which were recruited through existing relationships with the research team and one *via* Mencap, who provided a letter of support for the funding application. Each site delivered between one and three intervention group programmes. Facilitators were trained to deliver the intervention in a group setting, over 5 days by an E-PAtS trainer. Interviewed facilitators positively described the quality and depth of the training information provided, including scientific rationales, and methods of training used, resulting in participants feeling confident to deliver the programme: “*…it was quite in-depth so we went into quite a lot of psychology and the basis behind what makes the training work“ [Facilitator]*.

### Retention Through 3 and 12 Months' Post-randomisation Follow-Up Data Collection

At 3 months post-randomisation, 84% (*n* = 31) and 78% (*n* = 29) of intervention and control families, respectively, completed follow-up data, including either completion of the 5 min speech sample *via* telephone interview or questionnaire completion. At 12 months' post-randomisation, 76% (*n* = 28) and 68% (*n* = 25) of intervention and control families, respectively, completed follow-up data collection. Two families (three participants) withdrew from the trial between baseline data collection and 3 months' post-randomisation.

**Figure 1:**
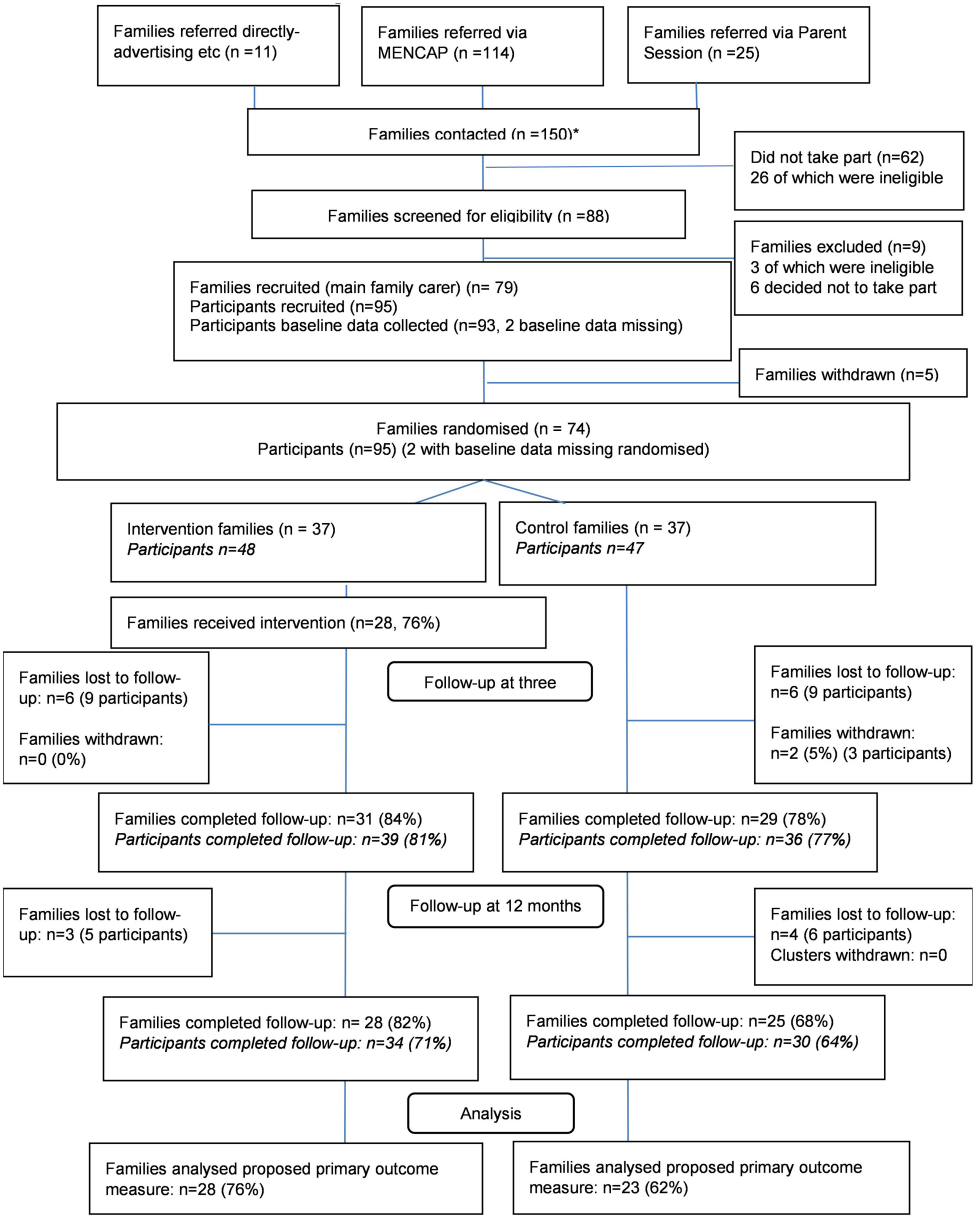
E-PAtS CONSORT diagram. ^*^A total of 150 families were contacted in the study. One of the sites recruited families in 2 rounds, and some of the families (n = 16) referred from this site were referred for both the first and second round (if they were not recruited on the first round).

Barriers to participation in follow-up data collection included unavailability due to holidays or family illness/hospitalisation. Furthermore, the 5 min speech sample requested that parents spoke about their thoughts and feelings about their child and their relationship with their child, without interruption. Interviewed participants described apprehension and difficulty completing the measure and response rates reduced from 100% at baseline to 62% at the 3 month follow-up. The 5 min speech sample was not repeated at 12 months' post-randomisation as it was not felt to be acceptable by a reasonable proportion of participants. Follow-up rates are presented in the CONSORT diagram (Figure 1) (36).

### Acceptability of Research Design

Interviewed participants described the research recruitment processes positively, including the provision of information about the trial, despite the perceived extensive, and complex information provided: “*There was a lot of information. Whatever we were told and whatever was happening at the time we were perfectly happy with, you know.” [Participant, control]*.

Randomisation in a 1:1 ratio to intervention or waitlist control group was generally acceptable to interviewed participants, despite some participants reporting that they found the information provided confusing: “*I think when I was initially putting my name down I just wanted to get the classes, but when it was explained I was happy enough. I mean, if everybody can't do it then everybody can't do it, and it was randomly selected.” [Participant, control]*.

Some interviewed participants also raised concerns about the requirement to wait 12 months to attend if they were randomised to the control arm. Some interviewed facilitators also felt that randomisation may have been a deterrent to participants: “*Yeah and I think the fact that it was the study with the control, that they might do it and they might not, I think that put people off.”[Facilitator]*.

Most participants chose the postal method at both 3 and 12 months' post-randomisation. Interviewed participants expressed that completion of the questionnaires was time-consuming and that this was off-putting: “*Long! Long! It was fine! The interviewer was very good and everything but it was long.” [Participant, control]*. Completion of the questionnaires was also found to be occasionally upsetting; participants acknowledged that self-examination, which was required to complete some questions, was difficult and also described experiencing a sense of realisation regarding the extent of their child's delay. However, some participants positively described completing the questionnaires with their partner and a resulting increased confidence and awareness of their child's needs: “*I think when you get a huge questionnaire like that it does bring it home to you, it's quite profound when you look at it in black and white and you see exactly the extent of the things you're dealing with which is different from your other children.” [Participant, intervention, main carer]*.

### The Acceptability of Intervention Delivery to Service Provider Organisations, Facilitators, and Family Caregivers Through Qualitative Interviews

Interviewed participants were overwhelmingly positive about the facilitators, they described the group sessions as informal and welcoming and they valued the peer support provided by other participants and the family carer facilitator: “*It was very intimate, everyone was very friendly, everyone was on first name terms - it was just appreciated.” [Participant, intervention, main carer]*. The delivery methods and session content, particularly the practical exercises, were typically received positively. Facilitators reported that the majority of participants were engaged and receptive.

### Adherence, Reach, and Fidelity of Implementation of the E-PAtS Intervention Through Attendance Records, Evaluation of Session Recordings, and Participant/Facilitator Qualitative Interviews

The majority of families adhered to the intervention (*n* = 27, 70.3%), defined as at least one family carer from a family attending at least five out of eight intervention sessions. Attendance ranged from 0 sessions (10.8%) to 8 sessions (18.9%), with a modal number of sessions attended by a family of seven (*n* = 11, 29.7%). Furthermore, exploratory Complier Average Causal Effects (CACE) analyses suggest that intervention adherence or increased attendance improved well-being, measured by the WEMWBS proposed primary outcome measure, at 12 months' post-randomisation (see Table 5). Reasons for non-attendance at group sessions, explored in participant interviews, included general challenges such as childcare requirements, family commitments, having to work, or travel difficulties.

**Table 5:** Between-arm differences on WEMWBS at 12-months post-randomisation with and without accounting for adherence or attendance.

**Model (47 family carers within 39 families)**	Adjusted mean difference^*^	**Lower 95% CI**	**Upper 95% CI**
Two-level model	3.96	−1.39	9.32
Single-level model^†^	4.38	−1.02	9.78
IV regression accounting for adherence^†^	5.05	−0.70	10.79
Maximum efficacy based on family session attendance	5.84	−0.80	12.40
Maximum efficacy based on main family carer attendance	6.84	−0.84	14.53
Maximum efficacy based on main family carer attendance, main family carer responses only (*n* = 36)	4.85	−3.71	13.41

* *Adjusted for baseline WEMWBS score and site*.

† *Cluster robust standard errors account for clustering of participants within families*.

Fidelity to the intervention, rated by the facilitators and from observational session recordings, was high. The mean proportion of items completed per session, reported by facilitators, was 97.1% (range 85–100%). The mean proportion of items observed to occur in sessions was 95.7% (range 88–100%). Out of a total 18 sessions randomly selected for observation across all sites however, only 7 sessions (38.9%) were video or audio recorded; 27.8% (*n* = 5) were not recorded due to technical issues, and at least one participant did not provide consent in 33.3% (*n* = 6) of sessions, potentially due to the sensitive nature of the discussions: “*because nobody wants their face in that, especially if they're talking about something so important and close to them”[Facilitator]*.

Interviewed facilitators were confident in their ability to deliver the sessions as planned, in part, due to the well-received facilitator training and associated training documents provided. If participants wanted to discuss topics not including in the course content, the facilitators felt comfortable doing so, if considered necessary. Barriers to intervention delivery included distractions when children were present and logistical issues such as poor internet connections.

### Usual Practise in this Setting and Use of Services/Support by Intervention and Control Participants

In a separate early intervention survey completed by the research group alongside the feasibility trial (4), 673 parents/family carers with a child with a diagnosed or suspected developmental disability, from across the UK, described any intervention of focused support they received for themselves or their child in the past 12 months. The majority of parents/family carers reported that they had not received any intervention or support (*n* = 476, 70.7%). Of the remaining 29.3% (*n* = 197), only 10.5% clearly named a parent training/support intervention (including Early Bird, Incredible Years parenting programmes, Stepping Stones Triple P, or therapy/counselling for themselves). Interviewed participants described E-PAtS as unique to other courses available, in part due to the co-delivery by a professional and carer facilitator aspect.

### Feasibility and Acceptability of Proposed Outcome Measures for a Definitive Trial, Including Resource Use and Health-Related Quality of Life Data

#### Participant-Reported Outcome Measures

Of the participant-reported outcome measures returned, a small number were unusable due to incomplete data completion, meaning that these could not be analysed for those participants. including: WEMWBS (*n* = 1, 12 months); family APGAR (*n* = 1, 3 months); positive gains scale (*n* = 1, 3 months); CPRS conflict (*n* = 2, baseline); CPRS closeness (*n* = 6, baseline; *n* = 3, 3 months; *n* = 4, 12 months); EQ-5D Visual Analogue Scale value (*n* = 1, 3 months; *n* = 1, 12 months); Brief COPE (*n* = 2, baseline; *n* = 1, 3 months); co-parenting agreement (*n* = 1, 12 months); CBCL (*n* = 5, baseline; *n* = 6, 3 months; *n* = 5, 12 months); SRQ (*n* = 9, baseline; *n* = 7, 3 months; *n* = 7, 12 months).

Interviewed participants described some barriers to completing outcome measures, including: specific questions were not applicable due to the age/developmental stage of the child, difficulties recalling required information, and response options not always being appropriate: “*…the answers didn't always fit. You felt that you were choosing the answer but really your answer needed a bit more nuance.”[Participant, control]*. The Child Behaviour Checklist was identified as being particularly difficult to answer due to the volume of questions and one participant questioned the relevance of the finance questions: “*Disability doesn't really discriminate against class... Similar questions have been asked of me, by therapists, but I did find it a bit intrusive.” [Participant, intervention, main carer]*.

Summary statistics for each outcome measure, for intervention and control groups, are presented in Table 6. The results from the exploratory multilevel regression analysis comparing the intervention and control groups, adjusting for baseline score and site, for each outcome measure are presented in Table 7. Although the feasibility RCT was not powered to detect effectiveness (37), the effect sizes of all of the outcome measures, with the exception of the “count of formal sources” section of the Family Support Scale are suggestive of positively favouring the intervention group. WEMWBS is the proposed primary outcome for a definitive trial. A higher score for WEMWBS indicates improved well-being (the mean UK norm is 51). Ninety-five percent of participants completed WEMWBS at baseline, all to a useable extent (*n* = 90). Forty-nine percent of control arm participants (*n* = 23) and 56% of intervention arm participants (*n* = 27) completed the measure at the 12-month time-point, again all to a usable extent. In the control group, the mean WEMWBS score was 43.2 (SD = 8.9) at baseline and 43.4 (SD = 11.0) at 12 months' post-randomisation. In the intervention group, scores were 43.9 (SD = 10.6) and 46.5 (SD = 10.9), respectively. After adjusting for baseline WEMWBS scores and variables balanced on at randomisation, the mean WEMWBS score at 12-months post-randomisation was 3.96 points higher in the intervention arm compared to the control arm (95% CI: −1.39 to 9.32 points).

**Table 6:** Proposed participant-reported outcome measures by trial arm for all participants.

**Measure**		**Control arm**			**Intervention arm**		
		**Time point**			**Time point**		
		**Baseline**	**3 month**	**12 month**	**Baseline**	**3 month**	**12 month**
Warwick-Edinburgh Mental Well-Being Scale (WEMWBS)–score range 14–70, higher scores indicate higher levels of mental well-being	Mean (sd)	43.2 (8.9)	42.7 (9.4)	43.4 (11.0)	43.9 (10.6)	45.5 (9.2)	46.5 (10.9)
	Range	23–62	21–60	21–65	19–66	23–61	25–68
Hospital Anxiety and Depression Scale (HADS) Anxiety–score range 0–21, high scores indicate greater anxiety	Mean (sd)	10.6 (3.7)	11.4 (4.8)	9.9 (4.4)	10.1 (4.4)	10.2 (4.9)	8.0 (4.4)
	Range	0–19	4–20	3–19	1–21	1–19	1–20
Hospital Anxiety and Depression Scale (HADS) Anxiety Depression–score range 0–21, high scores indicate higher levels of anxiety	Mean (sd)	7.9 (3.9)	8.6 (4.1)	8.9 (4.2)	7.2 (4.0)	7.1 (4.4)	6.1 (4.4)
	Range	0–16	2–20	3–20	0–17	0–15	0–14
Hospital Anxiety and Depression Scale (HADS) Anxiety Emotional distress–sum of anxiety and depression subscales–score range 0–42, high scores indicate greater emotional distress	Mean (sd)	18.4 (6.8)	19.9 (7.7)	18.8 (8.0)	17.3 (7.8)	17.2 (8.8)	14.1 (8.2)
	Range	0–33	6–32	6–34	4–38	1–32	1–32
Vineland Adaptive Behaviour Scales (Vineland-3)–child level variable. Adaptive Behaviour Composite (ABC) score–standardised score, mean 100.	Median (IQR)	55 (40, 67)	N/A	64.5 (58, 69)	58 (50, 66)	N/A	67.5 (58.5, 70.5)
	Range	25–78	N/A	46–73	34–76	N/A	45–73
Vineland Adaptive Behaviour Scales (Vineland-3)–child level variable. Communication sub-domain -standardised score, mean 100.	Median (IQR)	44 (26, 67)	N/A	63 (52, 70)	55 (34, 64)	N/A	61 (52, 70.5)
	Range	20–83	N/A	39–77	20–85	N/A	40–80
Family APGAR scale−5 items, score range 0–10. Higher scores indicate better family function	Mean (sd)	7.3 (2.4)	7.5 (2.7)	6.5 (3.2)	6.9 (2.9)	7.1 (3.0)	6.4 (2.8)
	Range	2–10	1–10	0–10	0–10	0–10	1–10
Family Support Scale-Number of informal sources of support available	Median (IQR)	10 (8, 12)	11 (9, 12)	11 (10, 13)	10 (7, 12)	8 (6, 11)	10 (7.5, 11)
	Range	5–13	3–13	2–13	3–13	2–13	2–13
Number of formal sources of support available	Median (IQR)	4 (3, 5)	4 (4, 5)	4 (4, 5)	4 (3, 5)	4 (3, 5)	4 (3, 5)
	Range	1–5	3–5	3–5	1–5	2–5	2–5
Mean helpfulness of informal sources of support available–scored 0 (not at all helpful)−4 (extremely helpful)	Mean (sd)	1.8 (0.8)	1.8 (0.7)	1.6 (0.6)	2.0 (0.9)	2.0 (0.8)	2.0 (1.0)
	Range	0.5–3.5	0.7–3.0	0.5–3.0	0.5–4.0	0–3.8	0.6–4.0
Mean helpfulness of formal sources of support available–scored 0 (not at all helpful)−4 (extremely helpful)	Mean (sd)	2.4 (1.0)	2.3 (0.8)	2.0 (1.1)	2.5 (1.0)	2.5 (0.9)	2.6 (1.0)
	Range	0.6–4.0	0.5–4.0	0.4–4.0	0.2–4.0	0.6–4.0	0.5–4.0
Positive Gains Scale−7 items, score range 7–35. Higher scores indicate higher positive gains	Median (IQR)	13 (9, 15)	12.5 (10, 15)	12 (9, 14)	11 (8, 15)	12 (9, 15)	11 (8, 14)
	Range	7–24	7–19	7–19	7–23	7–35	7–20
Child-Parent Relationship Scale (CPRS)−15 items. Conflict−8 items, score range 8-40, high scores indicate greater conflict	Mean (sd)	18.9 (6.4)	20.0 (6.1)	20.3 (6.2)	19.2 (6.8)	18.5 (8.0)	18.0 (7.3)
	Range	8–32	9–30	10–32	8–33	8–35	8–32
Child-Parent Relationship Scale (CPRS)–Closeness−7 items, score range 7–35, low scores indicate a less close relationship	Mean (sd)	25.9 (5.4)	25.8 (5.5)	27.6 (3.7)	26.9 (4.7)	28.1 (5.2)	29.7 (3.8)
	Range	13–35	11–34	19–35	17–35	15–35	22–35
Child-Parent Activity Index−5 items, score range 5–25. Higher scores indicate higher frequencies of activities shared with child	Mean (sd)	20.6 (3.4)	20.4 (3.3)	20.9 (3.1)	20.4 (3.1)	20.6 (3.4)	20.6 (3.2)
	Range	13–24	13–25	14–25	12–25	12–25	13–25
Brief COPE−17 items, 3 subscales. Active avoidance coping–score range 6–24	Mean (sd)	13.9 (3.1)	12.1 (2.4)	12.3 (2.7)	13.2 (3.5)	13.3 (3.4)	12.8 (3.4)
	Range	8–20	8–18	7–18	6–21	8–20	7–21
Problem focused coping–score range 5–20	Mean (sd)	18.8 (3.4)	18.2 (3.4)	18.2 (3.6)	18.0 (3.4)	18.8 (3.0)	19.2 (2.9)
	Range	11–24	10–23	11–24	10–24	11–24	14–24
Happiness of Relationship scale−1 item scored 1–7. Higher scores indicate greater happiness	Median (IQR)	6 (5, 7)	7 (5, 7)	6.5 (5, 7)	7 (6, 7)	6 (4.5–7)	6 (5, 7)
	Range	1–7	1–7	1–7	1–7	1–7	1–7
Co-parenting agreement−4 items, score range 0–6. Higher scores indicate greater co-parenting agreement	Median (IQR)	5.5 (4.3, 6.0)	5.6 (4.3, 5.8)	5.3 (4.8, 5.8)	4.8 (3.5, 6.0)	4.3 (3.5, 6.0)	5.0 (3.3, 6.0)
	Range	1.0–6.0	0.5–6.0	0.3–6.0	0.5–6.0	0–6	2.3–6.0
Conflict−1 item scored 1–7. Higher scores indicate greater exposure to conflict	Median (IQR)	2 (1, 3)	2 (1, 4)	2 (1, 3)	2 (1, 2)	2 (1, 4)	2 (1, 3)
	Range	1–6	1–6	1–6	1–4	1–6	1–4
Child Behaviour Checklist (CBCL) –Internalising score.	Mean (sd)	19.1 (9.0)	19.5 (11.0)	21.8 (11.6)	19.8 (11.3)	18.2 (12.8)	18.2 (13.6)
	Range	3–37	3–40	9–46	2–5121.	3–49	1–42
Child Behaviour Checklist (CBCL) –Externalising score.	Mean (sd)	21.5 (9.7)	22.6 (11.5)	22.9 (10.3)	19.0 (11.5)	17.6 (13.8)	16.6 (11.7)
	Range	3–42	2–43	4–41	2–44	0–46	1–46
Child Behaviour Checklist (CBCL) –Total problem score	Mean (sd)	67.5 (26.4)	70.6 (33.6)	73.2 (27.5)	63.3 (32.5)	59.2 (38.9)	56.8 (34.1)
	Range	13–120	7–129	16–120	10–142	9–140	3–115
Paediatric Quality of Life Inventory–Total score–score range 0–100, high scores indicate better health related quality of life	Mean (sd)	55.0 (16.9)	57.0 (18.6)	48.5 (21.2)	61.6 (17.6)	59.8 (17.7)	61.2 (17.3)
	Range	26–94	17–89	0–85	24–85	26–8,916	19–87
Group Cohesion Scale–score range 8–32, high scores indicate better group cohesion	Median (IQR)					29.5 (24.5, 32.0)	
	Range					8–32	
Strengths and Difficulties Questionnaire (for siblings)−25 items (higher scores indicate a higher degree of problems for each subscale)	Median (IQR)	8.0 (7.5, 9.5)	8.0 (7.0, 9.0)	7.5 (7.0, 9.0)	8.5 (6.0, 10.0)	8.5 (5.0, 10.0)	8.0 (6.0, 10.0)
	Range	1.2–10.0	5.0–10.0	1.0–10.0	1.0–10.0	0.0–10.0	5.0–10.0
Internalising problems–sum of emotional and peer problems subscale, score range 0–20	Median (IQR)	4.5 (2.5, 9.0)	4.0 (3.0, 7.0)	5.5 (4.0, 11.0)	2.0 (1.0, 9.0)	5.5 (2.5, 8.0)	4.0 (3.0, 6.0)
	Range	0.0–15.0	0.0–13.0	0.0–15.0	0.0–16.0	0.0–11.0	0.0–17.0
Externalising problems–sum of hyperactivity and conduct, score range 0–20	Median (IQR)	7.0 (3.0, 9.0)	5.0 (4.0, 9.0)	8.0 (3.0, 11.0)	4.5 (2.0, 7.0)	6.8 (3.5, 10.0)	4.5 (3.0, 7.0)
	Range	1.0–13.3	2.0–13.0	1.0–12.0	0.0–15.0	0.0–18.3	0.0–15.0
Sibling Relationship Quality (SRQ)	Mean (sd)	3.1 (0.8)	3.1 (0.6)	3.1 (0.7)	3.4 (0.5)	3.4 (0.9)	3.6 (0.7)
	Range	1.0–4.3	2.0–4.2	1.5–4.0	2.3–4.3	2.0–5.0	2.5–4.8
Conflict–score range 1–5, high scores indicate higher levels of conflict in relationship	Mean (sd)	2.0 (0.9)	2.2 (0.9)	2.4 (1.3)	1.9 (0.7)	2.1 (0.8)	2.1 (0.7)
	Range	1.0–4.0	1.0–4.5	1.0–4.3	1.0–3.3	1.0–3.5	1.0–3.0

*An additional 6 forms in total were erroneously completed by main family carers of children younger than 3 years old and are not recorded in these figures*.

**Table 7:** Two level regression analysis, comparing intervention to control group.

			**Two level model**	
**Measure**	***n***	**% of randomised**	**Estimate**	**(95% CI)**
WEMWBS	47	49.5	3.96	(−1.39 to 9.32)
HADS				
Anxiety	50	52.6	−1.62	(−3.39 to 0.15)
Depression	50	52.6	−1.30	(−2.89 to 0.28)
Total–emotional distress	50	52.6	−2.89	(−5.83 to 0.04)
Vineland-3				
Adaptive Behaviour Composite (ABC)	42	44.2	0.42	(−3.03 to 3.88)
Communication sub-domain	42	44.2	−1.17	(−6.83 to 4.50)
APGAR	50	52.6	0.49	(−0.90 to 1.88)
Family Support Scale				
Count informal sources	49	51.6	−0.82	(−1.94 to 0.29)
Count formal sources	49	51.6	−0.60	(−1.04 to −0.16)
Mean helpfulness, informal	49	51.6	0.15	(−0.24 to 0.55)
Mean helpfulness, formal	49	51.6	0.40	(−0.22 to 1.02)
Positive Gains Scale	47	49.5	0.18	(−2.06 to 2.41)
Child-Parent Relationship Scale				
Conflict	50	52.6	−0.78	(−3.89 to 2.32)
Closeness	45	47.4	0.60	(−1.33 to 2.53)
Child-Parent Activity Index	51	53.7	0.22	(−1.24 to 1.68)
Happiness of Relationship scale	42	44.2	0.33	(−0.51 to 1.17)
Co-parenting agreement scale	39	41.1	0.06	(−0.80 to 0.93)
Conflict	41	43.2	−0.12	(−1.13 to 0.89)
EQ-5D-5L				
EQ-VAS	50	52.6	1.70	(−5.81 to 9.22)
Index Value	50	52.6	0.04	(−0.04 to 0.12)
Brief COPE				
Active avoidance	50	52.6	0.46	(−1.14 to 2.06)
Problem focused	50	52.6	0.16	(−1.35 to 1.68)
Positive coping	50	52.6	0.52	(−1.12 to 2.22)
Child Behaviour Checklist (CBCL)				
Internalising score	41	43.2	−2.80	(−7.60 to 2.00)
Externalising score	41	43.2	−1.86	(−5.55 to 1.82)
Total problems	41	43.2	−9.00	(−20.79 to 2.88)
Paediatric Quality of Life Inventory (total score)	46	48.4	7.0	(−1.84 to 15.78)
Strengths and Difficulties Questionnaire–siblings (SDQ)				
Prosocial	32	33.7	0.5	(−1.07 to 2.17)
Internalising score	32	33.7	−1.6	(−4.32 to 1.12)
Externalising score	32	33.7	−0.6	(−3.47 to 2.37)
Sibling Relationship Questionnaire				
Warmth	19	20.0	0.1	(−0.63 to 0.84)
Conflict	25	26.3	−0.3	(−0.84 to 0.30)

#### Resource Use and Health-Related Quality of Life Data

The feasibility of conducting a health economic evaluation in a definitive trial was assessed. Costs were determined for two sites (sites 1 and 3). The mean intervention costs per session were £91.37 and £109.55 for sites 1 and 3, respectively, and initial site-wide training (set up) costs were £3174.17 and £3426.67 in sites 1 and 3, respectively.

The feasibility of collecting resource use data, including participants' and their child's use of healthcare services, hospital care services, children's development centres and children's day centres, community-based healthcare and medicine and legal and social services (for parent only) in a definitive trial was established. Resource inputs were valued using various secondary sources for unit costs [NHS Reference Costs Trusts schedules, (38) the Personal Social Services Research Unit cost compendium (39), NHS Reference Costs Trusts schedules (37), the Childcare costs survey (40), and the British National Formulary (BNF) or the British National Formulary for Children (BNFC) (41)]. 61.1 and 49.5% of randomised participants completed the resource use questionnaires at 3 and 12 months, respectively. Individual resource use questions were well-completed, but interviewed participants described difficulty in recalling some resource use/cost estimates over extended recall periods. All participants who completed the 5D-5L measure completed it to a usable degree.

The extraction of data from routine data sources in a future definitive trial would allow the validation of, or addition to, participant-reported resource use data. A later definitive trial may extract key resource use items from the Mental Health Minimum Data Set (MHMDS) (data regarding children and young people's access to psychological therapies, intellectual disabilities or autism services) and Hospital Episode Statistics (HES) (data detailing NHS hospital admissions in England).

The feasibility trial identified limitations in expressing the cost-effectiveness of the E-PAtS programme in terms of incremental cost per unit change in the WEMWBS proposed primary outcome measure. The WEMWBS is not currently a preference-based measure that permits the estimation of quality-adjusted life years (QALYs) amenable to cost-effectiveness decision-making (42). |The feasibility trial identified a number of attributes from the qualitative research that can potentially be incorporated into a discrete choice experiment (DCE), a preference-based approach for valuing potentially disparate effects of interventions. A DCE can be incorporated into a future trial-based economic evaluation of the E-PAtS programme with a view to informing a cost-benefit analysis.

### Acceptability of Collecting and Analysing Routine Data Within a Definitive RCT

Forty-seven participants (49.7%) answered questions regarding their views on the acceptability of collecting routine data in a future definitive trial. Less than 40% of participants were aware that researchers were able to access routine data from hospital records, school records, or social care records. The data suggest that participants in the intervention group were less comfortable than the control group with the idea of extracting their own or their child's routine data. Furthermore, 41% of intervention participants reported that requesting their hospital data would deter them from taking part in a trial and 42% reported that requesting their child's hospital data would deter them from participating. All responses are presented in Table 8. Interviewed participants' responses regarding the use of routine data in a definitive trial were varied; some participants were comfortable with the proposal whilst others stated that it would deter them from taking part. Clarity of the information collected, particularly in the social services data which was considered sensitive by participants, may lead to greater consent levels.

**Table 8:** Participants' awareness of and views surrounding routine data collection.

	**Hospital data-parent**		**Hospital data- child**		**School data**		**Social care data- child**	
	**Cont (*n* = 23)**	**Int (*n* = 24)**	**Cont (3*n* = 23)**	**Int (*n* = 24)**	**Cont (*n* = 23)**	**Int (*n* = 24)**	**Cont (*n* = 23)**	**Int (*n* = 24)**
	***n* (%)**	***n* (%)**	***n* (%)**	***n* (%)**	***n* (%)**	***n* (%)**	***n* (%)**	***n* (%)**
Were you aware researchers are able to request access to this data?								
No	13 (57)	16 (67)	N/A	N/A	17 (74)	17 (71)	16 (70)	17 (71)
Yes	9 (39)	7 (29)	N/A	N/A	6 (26)	7 (29)	7 (30)	7 (29)
Missing	1 (4)	1 (4)	N/A	N/A	0 (0)	0 (0)	0 (0)	0 (0)
Would you be comfortable in agreeing to us accessing this data in a future trial?								
Not at all comfortable	0 (0)	6 (25)	0 (0)	4 (17)	0 (0)	4 (17)	0 (0)	4 (17)
Not very comfortable	2 (9)	5 (21)	1 (4)	5 (21)	1 (4)	2 (8)	1 (4)	3 (13)
No preference	9 (39)	4 (17)	10 (43)	3 (13)	13 (57)	7 (29)	14 (61)	7 (29)
Quite comfortable	7 (30)	7 (29)	7 (30)	9 (38)	4 (17)	8 (33)	2 (9)	8 (33)
Very comfortable	4 (17)	2 (8)	4 (17)	3 (13)	4 (17)	3 (13)	5 (22)	2 (8)
Missing	1 (4)	0 (0)	1 (4)	0 (0)	1 (4)	0 (0)	1 (4)	0 (0)
Would it have affected your decision to take part in E-PAtS, if we had asked for consent to collect this data?								
Definitely less likely to take part	0 (0)	4 (17)	0 (0)	6 (25)	0 (0)	3 (13)	0 (0)	4 (17)
Slightly less likely to take part	2 (9)	5 (21)	3 (13)	4 (17)	2 (9)	1 (4)	2 (9)	3 (13)
No difference	18 (78)	12 (50)	17 (74)	12 (50)	17 (74)	17 (71)	17 (74)	15 (63)
Slightly more likely to take part	0 (0)	1 (4)	1 (4)	1 (4)	1 (4)	1 (4)	1 (4)	1 (4)
Definitely more likely to take part	2 (9)	0 (0)	1 (4)	1 (4)	2 (9)	1 (4)	2 (9)	1 (4)
Missing	1 (4)	2 (8)	0 (0%)	0 (0%)	1 (4)	1 (4)	1 (4)	0 (0)

### Service Provider Organisation Willingness to Participate in a Definitive Trial

Fifteen organisations, representative of those likely to be invited to provide E-PAtS within a future trial, responded to a survey distributed by email. Barriers to taking part in a future definite trial included: (1) concerns regarding the feasibility of securing additional/external funding for training and programme delivery; and (2) the need to consult with and gain approval from senior management. The majority of organisations (13/14 which answered the relevant question) indicated however, that they were somewhat or very likely to participate in a future RCT.

## Discussion

The objectives of the trial were to test the feasibility of evaluating E-PAtS in a definitive RCT. Recruitment of service provider organisations and facilitators was shown to be feasible, and trial design and intervention implementation processes, including facilitator training, were well-received. Service provider organisations were hesitant regarding randomisation in this setting, but were persuaded due to the perceived importance of the research, something that should be handled carefully when recruiting additional service provider organisations in a future trial. Participant recruitment was successful overall, albeit with site differences in recruitment rates. An additional recruitment period was required to meet the target, due to differences in school term times in one of the sites recruited to take part in the feasibility RCT. Successful recruitment pathways included those that involved families already known to the service provider organisations and therefore assessment of service provider organisations' existing links with potentially eligible families should be a consideration in a definitive trial.

Recruitment and data collection processes were generally acceptable to participants and follow-up rates were good, with 81 and 73% of families completing at least one outcome measure in the intervention and control group, respectively. However, completion of the proposed primary outcome measure was lower at 51% overall, but those that did complete the measure did so to a usable degree to allow statistical analysis (>98% usable). The trial team modified the package of outcome measures, by removing the 5 min speech sample at the 12 month follow-up due to negative reactions by a number of participants and a significant reduction in completion rates between baseline and the 3 month follow-up. However, feedback from participant interviews was that the questionnaires were long. Therefore, optimising and selecting the most appropriate outcome measures to be used in the package of measures for a definitive trial, plus focusing the primary outcome measure, may improve retention and primary outcome measure completion. The outcome measures were also designed to be comprehensive in covering potential effects of E-PAtS, and there is no expectation that such a questionnaire package of the current size would be used in later standard community or clinical delivery of the intervention.

Measures used for health economic evaluation (the health-related quality of life measure (EQ-5D-5L) and resource use questions) were completed to a high degree. Although participants reported that some resource use questions were difficult to answer due to recall, the results suggest that a health economic evaluation would be feasible in a definitive trial. A future trial is needed in which the question of cost-effectiveness is examined in detail. It was also possible to cost the intervention delivery at two sites involved in the study. However, more data would be required before a likely range of costs for the intervention delivery could be determined to reliably inform services considering using E-PAtS.

The results suggest that although extraction from routine data sources would be acceptable in a definitive trial, clear educational messaging for participants, detailing exactly what data would be extracted, would be essential to alleviate participants' concerns and the chance that potential participants may not participate due to a routine data component. In addition, the trial team would need to consider the barriers to routine data collection including: extended time to collect data, due to the time required to process applications [82], and ethical and legal approvals required; cost implications; and limitations collecting social care data from routine data sources. NHS Digital (England), Mental Health Services Data Set (MHMDS), and the National Pupil Database (NPD (England) are all potential data sources of interest for a future trial. If included in the definitive trial, logistical considerations will need to be incorporated into the final design, including ensuring: (1) appropriate consent; (2) collection of data to allow data matching for all routine data sources; (3) data linking across all providers; and (4) that data storage requirements are followed.

The intervention sessions were perceived positively, with parents/family carers valuing the group aspect of the E-PAtS sessions and peer support. However, the current study was not designed to provide a critical examination of the content of E-PAtS either from the perspective of families taking part or the perspective of facilitators. The co-production process with family carers and expert professionals ensures that the content brings together theory and evidence-based practise with families' direct experience. However, before a definitive trial of E-PAtS is conducted a thorough review of the intervention and revision of its underlying Logic Model should be carried out.

Preliminary analysis of outcome measures, including the primary outcome measure WEMWBS, suggested that at 12 months, scores were in a favourable direction, demonstrating the expected changes that are described in the logic model. Adherence, as defined as family attendance at five of the eight intervention sessions, was relatively high (76%), but with only the main family carer attending most sessions. Facilitator-reported and observer-measured fidelity ratings demonstrated that the intervention group sessions were delivered to a high standard across all sites, suggesting that adequate training and support were provided to the facilitators and service provider organisations. In addition, exploratory analyses demonstrated that at 12 months, WEMWBS scores were higher in families that adhered to the intervention and attended more sessions. These results are encouraging of testing the effectiveness of E-PAtS in a future trial.

Finally, investigations into usual practise, in the form of a separate large survey and qualitative interviews with trial participants, demonstrated that support for parents with a young child with ID is perceived as unsatisfactory, with less than one third of parents/care-givers reporting receiving an appropriate parenting intervention in the preceding 12 months. E-PAtS therefore provides an opportunity to provide a unique (co-delivery with a parent/family carer facilitator), well-received intervention to this population.

## Strengths/Limitations and Future Research

Whilst most second family carers who did attend were fathers, only a small proportion of the main family carers were fathers, as is commonly the case with studies of families of children with ID (43). Fathers of young children with ID are twice as likely to score above the cut-off on a psychiatric disorder screen when compared to fathers of other young children (3). Therefore, a definitive RCT may benefit from focused recruitment of fathers in particular.

The majority of recruited parents/family carers reported having some higher education, but there was evidence of socio-economic deprivation in the sample (up to one half of family carers indicated that they were just managing or were struggling with finances and reported family weekly incomes were fairly low; 69% of main family carers reported a weekly household income of < £600). There was also limited ethnic diversity; 80% of all participants were white. Therefore, although the recruited sample did capture some diversity, to target less represented groups in a larger definitive RCT, some revisions to the recruitment methods, and additional PPI work will be required.

Due to the sensitive nature of the group sessions, some parents/family carers did not provide consent for E-PAtS sessions to be recorded and there were some technical difficulties, resulting in fewer than planned group sessions assessed for fidelity by the trial team. In those sessions that were recorded, however, observer-rated fidelity was high and similar to that reported by facilitators.

Finally, for a definitive RCT, intervention funding would need to be secured. Feedback from potential service provider organisations suggest that this may prove to be a barrier to conducting a future trial.

## Conclusions

The E-PAtS Feasibility RCT has demonstrated that the research design and methods of intervention implementation were generally feasible. Progression criteria, defined at funding application stage, were met and the independent Study Steering Committee approved progression to a future definitive trial. The limitations of this feasibility trial and barriers to conducting a future definitive trial need to be considered before progressing. Such a future trial is likely, as was the case for this feasibility study, to focus on effectiveness since a larger number of delivery sites in the community would be needed and this is unlikely to be associated with the opportunity for the programme developers to maintain tight control over the intervention delivery as would typically be the case in an efficacy trial.

## Data Availability Statement

The raw data supporting the conclusions of this article will be made available by the authors, without undue reservation.

## Ethics Statement

The studies involving human participants were reviewed and approved by University of Warwick Humanities and Social Sciences Research Ethics Committee. The patients/participants provided their written informed consent to participate in this study. All participants gave their consent for their pseudonymised data to be published in a research article.

## Author Contributions

EC managed the trial, conducted qualitative analysis, wrote up results for publication, and prepared the article for publication. NG co-led the trial and leads on the E-PAtS intervention. GM managed the trial and contributed to article writing. MW carried out the statistical analysis and wrote up the results for publication. JS led the process evaluation and wrote up results for publication. DG managed the statistical analysis and contributed to article writing. SP managed the health economics analysis and contributed to article writing. FL-W managed the linkage with routine data aspect and contributed to article writing. SK carried out the health economics analysis and wrote up results for publication. JB provided expertise in qualitative design and co-leads on the E-PAtS programme, the Vineland Adaptive Behaviour Scale analysis, and contributed to article writing. RM supervised the Cardiff University team and provided expertise in trial design and management. AJ provided expertise in qualitative methods and analysis and intellectual disability. GL provided expertise in parenting research. JS led the PPI component of the trial. VT provided expertise in parent involvement. CS carried out quantitative and qualitative data collection, carried out qualitative data analysis, and contributed to article writing. SF carried out quantitative and qualitative data collection and carried out qualitative data analysis. AC carried out quantitative and qualitative data collection and carried out qualitative data analysis. CB carried out data management. RH co-led the trial and prepared the article for publication. All authors contributed to article writing, read, and approved the final manuscript.

## Funding

This project was funded by the National Institute for Health Research (NIHR) Public Health Research programme (PHR/15/126/11). The funder had no role in trial design, data collection, analysis, interpretation, or preparation of this article.

## Author Disclaimer

The views expressed are those of the authors and not necessarily those of the NIHR or the Department of Health and Social Care. The project will be published in full in Public Health Research.

## Conflict of Interest

NG is programme developer for the E-PAtS intervention and has a patent Intellectual Property and copyrighted materials for E-PAtS. NG and JB receive fees for training in the E-PAtS intervention. RH collaborates with NG on other E-PAtS research. The remaining authors declare that the research was conducted in the absence of any commercial or financial relationships that could be construed as a potential conflict of interest.

## Publisher's Note

All claims expressed in this article are solely those of the authors and do not necessarily represent those of their affiliated organizations, or those of the publisher, the editors and the reviewers. Any product that may be evaluated in this article, or claim that may be made by its manufacturer, is not guaranteed or endorsed by the publisher.

## Abbreviations

ABC: Adaptive Behaviour Composite
BNF: British National Formulary
BNFC: British National Formulary Children
CACE: Complier Average Causal Effects
CBCL: Child Behaviour Checklist
CBF: Challenging Behaviour Foundation
CONSORT: Consolidated Standards of Reporting Trials
CPRS: Child Parent Relationship Scale
CTR: Centre for Trials Research
DCE: Discreet Choice Experiment
E-PAtS: Early Positive Approaches to Support
EQ: EuroQol
HADS: Hospital Anxiety and Depression Scale
HES: Hospital Episode Statistics
ID: Intellectual disability
IQ: Intelligence Quotient
MHMDS: Mental Health Minimum Data Set
MRC: Medical Research Council
NHS: National Health Service
NICE: The National Institute for Health and Care Excellence
NIHR: National Institute for Health Research
NPD: National Pupil Database
PPI: Public Participant Involvement
QALY: Quality Adjusted Life Years
RA: Research Assistant
RCT: Randomised Controlled Trial
SD: Standard deviation
SPIRIT: Standard protocol items: recommendations for interventional trials
SRQ: Sibling Relationship Quality
UK: United Kingdom
UP: Usual practise
VABS: Vineland Adaptive Behaviour Scales
WEMWBS: Warwick Edinburgh Mental Well-Being Scale.
